# *Malassezia* Infections in Humans and Animals: Pathophysiology, Detection, and Treatment

**DOI:** 10.1371/journal.ppat.1004523

**Published:** 2015-01-08

**Authors:** Aristea Velegraki, Claudia Cafarchia, Georgios Gaitanis, Roberta Iatta, Teun Boekhout

**Affiliations:** 1 Mycology Research Laboratory, Microbiology Department, Medical School, National and Kapodistrian University of Athens, Athens, Greece; 2 Department of Veterinary Medicine, University of Bari Aldo Moro, Bari, Italy; 3 Department of Skin and Venereal Diseases, Faculty of Medicine, School of Health Sciences, University of Ioannina, Ioannina, Greece; 4 CBS-KNAW Fungal Biodiversity Centre, Utrecht, The Netherlands; 5 State Key Laboratory of Mycology, Institute of Microbiology, Chinese Academy of Sciences, Beijing, China; 6 Shanghai Key Laboratory of Molecular Medical Mycology, Changzheng Hospital, Second Military Medical University, Shanghai, China; Duke University Medical Center, United States of America

## Introduction

The fungal genus *Malassezia* comprises lipid-dependent and lipophilic yeast species that are part of the normal skin microbiota [1]. The 14 species are classified in class Malasseziomycetes in the Ustilaginomycotina of Basidiomycota [2]. *Malassezia* species can be involved in skin disorders, such as pityriasis versicolor, seborrheic dermatitis, atopic eczema, and folliculitis, and occur at higher population densities on scalps with dandruff than on scalps without dandruff [3], [4]. Occasionally, invasive infections by *Malassezia pachydermatis* and lipid-dependent *Malassezia* spp. occur in neonates, most often in those who are receiving intravenous lipid supplementation, or in immunocompromised patients receiving parenteral nutrition via a catheter. *Malassezia* spp. have not yet been cultured from the environment, but metagenomics identified *Malassezia* phylotypes from terrestrial and marine habitats [5]. For instance, *Malassezia* ribosomal DNA (rDNA) has been reported from soil nematodes [6], sponges [7], and rocks [8]. Undeniably, much remains to be discovered about the spectrum of habitats exploited by *Malassezia* that would advance our knowledge on the ecological relationships between the *Malassezia* skin biotic community, their hosts, and the environment. The aim of this article is to review and discuss the literature available on the pathogenesis, detection, typing, and treatment of *Malassezia* infections in humans and animals.

## Pathophysiology on Human Skin

**Figure 1:**
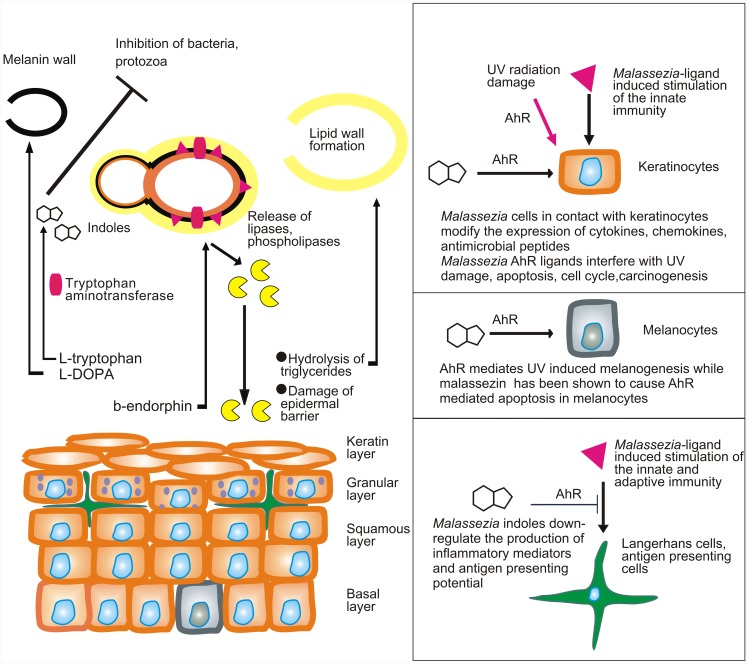
Model showing the putative interactions of *Malassezia* yeasts with the skin. *Malassezia* yeasts take up nutrients as well as sebum lipids that are used to form the outer layer of the yeast or amino acids that are needed for the formation of melanin or the synthesis of AhR indolic ligands. In parallel they modify the expression of lipases and phospholipases under the action of β-endorphin. Cellular components (enzymes, proteins, glyceroglycolipids, and mannosyl fatty acids) are recognized by the innate and adaptive immune system and alter its function. AhR ligands potentially down-regulate immune stimulation, modify the function of epidermal cells, interfere with AhR-induced ultraviolet (UV) damage and melanogenesis, and probably inhibit antagonist microbes.

The pathophysiology of *Malassezia*-caused or *Malassezia*-exacerbated skin conditions is largely unknown, owing to the complex interactions of this commensal with the skin, an organ that has been on the edge of extreme selection pressure during evolution. In healthy skin, *Malassezia* yeasts exploit essential nutrients for their growth without inflicting disease (Fig. 1). When this process is perturbed, *Malassezia* yeasts adapt by modifying the expression of enzymes involved in the acquisition of energy, such as lipases and phospholipases [9], [10], and at the same time synthesize an array of bioactive indoles that act through the aryl-hydrocarbon receptor (AhR), which is expressed on almost all cell types found in the epidermis [11].

A major challenge would be to comprehend the multifaceted interactions of *Malassezia* yeasts with the human skin during health and disease. These include (a) commensalism (healthy skin), as there is no strong evidence for a mutualistic or beneficial relationship of the *Malassezia* microbiome and the skin; (b) subtle alterations in the function of skin melanocytes, resulting in hypo- or hyperpigmented plaques with characteristic clinical absence of inflammation and mild alterations in the epidermal barrier function (*pityriasis versicolor*) (Fig. 1); (c) inflammation without generation of antibody-mediated immunity (seborrheic dermatitis and dandruff); (d) induction of specific immunity (atopic dermatitis); and (e) invasion and inflammation of the hair follicle (*Malassezia* folliculitis). Interestingly, the high lipase activity of *M. globosa* from folliculitis specimens during the summer months may be promoted by sweat components [12], such as sodium chloride and lactic acid, thus laying a framework for examining potential metabolome, structure and function relationships between *M. globosa* lipases and the human skin. In seborrheic dermatitis and dandruff, there is a difference in the quality of sebum lipids between healthy and diseased skin [13], while the expression and function of *Malassezia* lipases in addition to barrier function defects and individual susceptibility take part in the exacerbation of these conditions [14], [15]. Recently, culture and biopsy evidence supported an association of *M. restricta* and *M. globosa* with rare nipple hyperkeratotic lesions [16] in young women, who responded to a combination therapy of oral itraconazole and topical ketoconazole. This denotes that the metabolome of strains involved in rare presentations of skin diseases should be thoroughly investigated, clearly in conjunction with key host and environmental factors.

In that respect, at least two *Malassezia* yeast metabolic pathways, i.e., phospholipase production [17], [18] and indole pigment synthesis, have been associated with strains isolated from human and animal diseased skin. *Malassezia* produces potent indolic AhR ligands, such as indirubin and indolo [3,2-b] carbazole (ICZ) [19], which potentially modify the function of almost all cells found in the epidermis and express this receptor (Fig. 1). In view of the AhR participation in (a) carcinogenesis, (b) immune regulation, and (c) the mediation of ultraviolet radiation damage, a hypothesis on the potential contribution of *Malassezia* yeasts in skin carcinogenesis has been formulated [20].

## Risk Factors for *Malassezia* Fungemia and Disseminated Disease

Patients under total parenteral nutrition (TPN) and immunocompromised patients with increased length of stay (LOS) in intensive care units are at risk for *Malassezia* infections. Risk for *Malassezia* infections is also high in very-low-birth-weight infants (<1500 g) and highest in premature infants [21]. The mechanism of transmission to the infant is vertical or horizontal [22]. After host exposure, the degree of prematurity, the corresponding skin condition, endotracheal intubation, central vascular access, diseases such as necrotizing enterocolitis or focal bowel perforation, and abdominal surgery contribute to colonization. Colonization is further enhanced by the pathogen's virulence factors, including adherence properties that favour colonization and proliferation followed by biofilm formation in central vascular catheters [23], [24]. These, in conjunction with iatrogenic factors, comprising invasive treatments and use of broad-spectrum antibiotics, parenteral nutrition, and administration of postnatal steroids and gastric acid inhibitors, contribute to the infection processes [23]. Compromised or immature host immunity, delayed diagnosis followed by persistent *Malassezia* fungemia and subsequent delayed vascular catheter removal, tissue or valve injury, insufficient antifungal dosing, or coinfection may lead to dissemination and occasionally result in poor prognosis.

## Risk Factors for Otitis and Dermatitis in Animals

*M. pachydermatis*, normally present on the skin and in the ear canal of dogs and cats, frequently causes dermatitis and otitis in mammals. However, the pathogenic role of *Malassezia* in the occurrence of lesions seems to be related to the host immune system as well as to yeast virulence factors [25], [26]. Particular conditions, such as atopic or seborrheic dermatitis, parasitic infestation, diabetes mellitus in dogs, feline immunodeficiency virus, and feline leukaemia virus infections, and long-term antibiotic use associated with glucocorticoid treatment may predispose to *Malassezia* overgrowth, usually leading to development of lesions [27]. Additionally, lesions might appear as a consequence of hypersensitivity reaction to yeast allergens or might be prevented by active stimulation of the reticuloendothelial system, as previously shown in dogs infected with *Leishmania* spp. [25].

The zymogen proenzyme of the yeast cell wall may activate the complement system, instigating damage to keratinocyte integrity, and lead to epidermal spongiosis, inflammation, and pruritus. Additionally, the yeast produces esterase, lipase, phosphatase acid, lipoxygenase, protease, and phospholipase enzymes that are recognized as virulence factors [17], [28].

Further studies demonstrated that the expression of phospholipase in *M. pachydermatis* is modified by the endogenous opioid peptide β-endorphin [29], and this is mediated from mu-opiod receptors that are present on the cell wall of this species [30]. Thus, these receptors seem to impact the phenotype (commensal or pathogenic) of this species under the action of appropriate agonists (β-endorphin) or antagonists (naloxon) [30]. The pathogenic role of *Malassezia* yeasts seems to be related to changes in the chemical or immunological mechanisms of the skin, which may modify the composition of the *Malassezia* cell wall. The recently elucidated polysaccharide organization of the *M. restricta* cell wall showed that this is unique among the fungi with an average content of 5% chitin, 20% chitosan, 5% **β**-(1–3)-glucan, and 70% **β**-(1–6)-glucan that form a large alkali-insoluble complex [31].

## Detection of *Malassezia* Infections in Humans and Animals

Isolation and enumeration of *Malassezia* cells from clinical specimens remains a challenge because of their lipid dependency. Since the clinical features, laboratory markers, and strategies for patient management do not differ between *Candida* and *Malassezia* fungemia, a more accurate etiological diagnosis is needed in high-risk patients by employing lipid-supplemented culture media in the current mycological routine [21], [32]. For septicaemia, contemporary paediatric aerobic lysis and centrifugation bottles supporting the growth of this yeast are recommended [32], followed by subculturing on lipid-supplemented media, such as modified Dixon or Leeming and Notman agars. Use of Sabouraud dextrose agar with the addition of a few drops of sterile olive oil does not support growth of all *Malassezia* spp. [33].

In contemporary clinical diagnostics, negative blood culture results obtained by the *Candida* QuickFISH BC platform (AdvanDx, Massachusetts, United States) for common yeasts and presence of the typically flask-like yeast cells in the gram-stained blood culture smear suggest possible infection by *Malassezia* spp., thus prompting employment of appropriate management and control strategies. Commercial molecular assays promise rapid and reliable detection of bloodstream and invasive *Malassezia* infections. Broad-spectrum real-time PCR platforms may prove useful tools for direct detection of *Malassezia* spp. in clinical specimens. The SeptiTest assay (Molzym, Bremen, Germany), engaging PCR followed by amplicon sequencing, can detect and discriminate *M. furfur* DNA in spiked clinical specimens with an analytical sensitivity corresponding to 2.5–3.5 *M. furfur* genomes per sample (A. Velegraki, unpublished data), as estimated based on the pulsed-field gel electrophoresis (PFGE)–generated *M. furfur* genome size range of 8.5–14 Mb [34]. Nonetheless, this assay needs validation for clinical use.

*Malassezia* species colonize a wide range of animals but may also cause disease to them [35]–[37]. *Malassezia* dermatitis is suspected in animals with inflammatory skin diseases characterized by erythematous or greasy lesions, especially in the intertriginous areas [36], [37]. As in humans, techniques for direct microscopy include the impression of cotton swab samples on glass slides and/or adhesive tape strips. Cultures are performed by inoculating specimens collected by cotton swabs or directly by contact plates containing lipid-supplemented media [26], [27]. Microscopy of swab specimens is useful for diagnosing animal and human dermatitis. The presence of ten or more yeast cells in five fields at 40× magnification from ear specimens of dogs indicate *Malassezia* otitis, whereas the presence of five cells from skin specimens suggests dermatitis [27]. Cultures are required only when direct microscopy is negative in animals with suspected infections. In this case, more than 70 colony-forming units per sample might be indicative of infection when the sample was collected by swabbing an area of 25 cm^2^ [27].

## Identification and Genotyping

Identification of *Malassezia* isolates can to some extent be achieved by microbiological and physiological assays [32], [33], [38]. However, molecular diagnostic methods are preferred for both strain identification and typing. These may comprise PCR–restriction fragment length polymorphism (RFLP) analysis of the internal transcribed spacer 2 (ITS2) region of rDNA, sequence analysis of the ITS 1+2 regions (including the 5.8S rRNA gene) of rDNA, the 5′ end of the large-subunit (LSU or 26S) rDNA, and the β-tubulin gene, and terminal fragment length polymorphism analysis (tFLP) [4], [23], [26], [28], [39]–[41]. Recently, matrix-assisted laser desorption/ionisation time-of-flight (MALDI-TOF) mass spectrometry (MS) has been used to identify *Malassezia* isolates [42], [43]. Direct identification and quantification of *Malassezia* species from specimens obtained from skin by adhesive transparent dressings using multiplex real-time PCR [44] also provides reliable identification outcomes.

The unravelling of *Malassezia* biodiversity in 1996 [45] was followed by enthusiasm concerning the possible association of particular species with skin diseases; however, this was not confirmed by experimental data. Subsequent genotyping studies in conjunction with conventional identification methods displayed a certain degree of concordance regarding (a) geographic origin and specific *Malassezia* spp. genotypes, (b) the relationship between particular genotypes and certain skin conditions, and (c) the correlation of *Malassezia* spp. genotypes with host species. A number of worldwide epidemiological studies indicated that *Malassezia* species may have distinctive geographic associations. This is highlighted by higher frequencies of *M. dermatis* and *M. japonica* in East Asia, compared with their rarity in the rest of the world [35], [46], [47]. Recently, *M. japonica* was reported from lesional and non lesional psoriasis patients' skin in India, thus expanding the geographical distribution of this species to South Asia [48]. Multiple genotypes and subgenotypes occurring on skin showed distribution patterns related to the host geographical origin [46] and to host skin sites with a specific microenvironment [26], [28], [49]. Amplified fragment length polymorphism (AFLP) analysis confirmed geographical variability among *M. furfur* isolates from Southeast Europe compared with those from other European regions. Sequence analysis of the intergenic spacer (IGS1) distinguished specific *M. globosa*, *M. restricta*, and *M. pachydermatis* variants in seborrhoeic dermatitis, atopic eczema, and on healthy skin. Moreover, sequence analyses of the LSU rDNA showed distinct *Malassezia* spp. subtypes from different host species [23]. Sequence analysis of chitin synthase 2 (*chs*2) indicated that clinical isolates of *M. pachydermatis* from cats and dogs cluster in four distinct genotypes (i.e., A, B, C, and D) linked to skin lesions or otitis.

Multilocus sequence analysis that included the D1/D2 domains of LSU rDNA, the *chs*2 gene, and the ITS1 region grouped *M. pachydermatis* strains from skin of healthy dogs and from skin lesions in three main genotypes (A, B, and C) with eight ITS1 subtypes [28], [40]. Genotype B included isolates from dogs of European origin and appears to be present on healthy dog skin, without producing phospholipase. The A and C genotypes and their subtypes seem to be predominantly associated with skin lesions and showed high phospholipase activity [28]. Similarly, IGS1 subtypes 3C and 3D displaying high phospholipase activity are more frequently isolated from skin lesions of dogs with atopic dermatitis [48].

A range of skin microenvironmental factors, such as the bacterial microbiota present, pH, salts, immune responses, biochemistry, and physiology, may play a role in adherence and growth of *Malassezia* species, favouring distinct genotypes depending on the geographical area and/or the skin sites [26], [40]. In addition, the biochemical composition of the skin selecting genetic populations of *Malassezia* yeasts can indirectly affect their drug susceptibility [50]. Indeed, *M. pachydermatis* genotype B, growing on skin enriched with lipids (i.e., healthy skin) showed lower fluconazole and higher ketoconazole, voriconazole, and posaconazole susceptibility than *M. pachydermatis* genotypes A and C [50]. Finally, the finding of different *Malassezia* genotypes or subtypes on distinct skin sites suggests that *Malassezia* yeasts may have a sexual or parasexual reproductive phase that might enhance its virulence, thus influencing the association between *Malassezia* spp. genetics and disease [28], [49], [51]. This possibility is strongly supported by the finding of mating type loci in both *M. globosa* and *M. sympodialis* [9], [10], [52].

## Susceptibility Testing and Treatment

*Malassezia* systemic infections require prompt identification of the pathogenic agent, removal of the central venous catheter and discontinuation of lipid supplementation, and treatment with liposomal amphotericin B [23], [53]. On the contrary, topical antifungal agents are adequate for the management of localized skin disease, while extensive disease requires administration of systemic itraconazole or fluconazole [23], [54]. This is also suitable for *Malassezia* folliculitis with concomitant modification of predisposing factors such as occlusion or systemic immunosuppresion. For the characteristic inflammatory conditions, seborrheic and atopic dermatitis, the addition of local anti-inflammatory therapy (i.e., corticosteroids or calcineurin inhibitors) is a prerequisite for rapid and effective control of exacerbations. One should always bear in mind that *Malassezia* yeasts are integral components of the skin microbiota and therefore the therapeutic target should be controlling the *Malassezia* population with subsequent long-term antifungal treatment, rather than eradicating it. Likewise, the need for extended (>2 months) azole treatment is required for suppression of symptoms in the *Malassezia*-triggered head and neck variant of atopic dermatitis [55]. Although the *in vitro* susceptibility testing is not yet standardized for *Malassezia* spp., the Clinical and Laboratory Standards Institute (CLSI) broth microdilution protocol was adapted by modifying media, time of incubation, and inocula, showing that itraconazole, ketoconazole, and posaconazole are the most effective drugs [50], [56].

*Malassezia* infections in animals are frequently treated with topical and/or systemic azole antifungal drugs [36]–[38], [57]–[59] usually combined with antibiotics and glucocorticoids in dogs with otitis externa [37], [38]. The emergence of azole-resistant *M. pachydermatis* [57], [58], as well as the increasing number of *Malassezia* infections in both humans and animals, emphasizes the importance of susceptibility tests as a guide for proper antifungal treatment [56].

Alternative therapeutic protocols, i.e., desensitization to *Malassezia* by immunotherapy or administration of inhibitors of yeast adherence factors, have been proposed to avoid repeated administration of antifungals and the occurrence of drug resistance phenomena [60]. Recently, the daily administration (150 µl, 2 mg/ml for 8 days) of a killer decapeptide, engineered from the variable region of a single-chain recombinant anti-idiotypic antibody, was shown to be a safe and effective treatment for *Malassezia* otitis externa in dogs [60].

## Conclusions

Over the last few decades, advances in research and technologies have greatly contributed to elucidating the role of *Malassezia* species in human and animal skin diseases and in human bloodstream infections. Molecular and alternative approaches have provided insights into the identification, taxonomy, and epidemiology of *Malassezia* species. In particular, PCR-RFLP, random amplified polymorphic DNA (RAPD), AFLP, PCR-single strand conformation polymorphism (SSCP) analysis, multilocus sequence typing (MLST, e.g., of ITS, IGS, *chs*2, and RNA polymerase 1 and 2), and MALDI-TOF MS resulted in the accurate identification and genotyping of *Malassezia* strains from humans or animals, thus resolving questions related to the geographical distribution of the infection agents and the characterization of strains causing outbreaks [61], [62]. Nevertheless, these studies showed that the diversity within a single *Malassezia* species can more likely be attributed to a high degree of evolution driven by ecology, host adaptation, and pathogenicity. In particular, the pathogenic role of *Malassezia* yeasts seems to be related to changes in the normal physical, chemical, or immunological processes in the skin, which may enhance or down-regulate the molecular production of yeast virulence factors or antigens [23], [39]. The chemical composition of host epidermis seems to play a pivotal role in influencing the pathogenic or commensal phenotype of *Malassezia* yeasts by selecting different genetic populations with specific physiological requirements, different cell wall compositions, and different antifungal susceptibility profiles. In addition, molecular and physiological studies suggest the possibility of sexual or parasexual reproduction that might have a role in the process of adaptation of different *Malassezia* genotypes on different hosts or skin sites. As a consequence, antifungal therapy in *Malassezia* infections requires careful appraisal of drugs chosen, especially in cases of unresponsiveness to the treatment or recurrent infections. So far, restoring the epidermal-barrier function and avoiding immunoglobulin E (IgE) sensitization seems to be useful for the prevention and treatment of skin diseases complicated by *Malassezia* [63], even if antifungal therapy remains the main effective treatment in the near future. Alternative future treatments seem to be the use of selected cell-penetrating peptides that are harmless for mammalian cells but have antifungal activity, as shown for *Malassezia* otitis in dogs [60].

Undoubtedly, proteomic and genomic studies are needed in order to better understand the relationship between particular species/genotypes of *Malassezia* and the host at molecular and biochemical levels. Detailed biochemical analysis of the cell wall of the various species, as recently performed for *M. restricta* [31], and studies on the genotypic variants and their interaction with the immune system seem important here. Such studies might be the base for designing methods for the prevention, treatment, and control of infections caused by these fungi.
